# Reducing FLI1 Levels in the MRL/lpr Lupus Mouse Model Impacts T Cell Function by Modulating Glycosphingolipid Metabolism

**DOI:** 10.1371/journal.pone.0075175

**Published:** 2013-09-10

**Authors:** Erin Morris Richard, Thirumagal Thiyagarajan, Marlene A. Bunni, Fahmin Basher, Patrick O. Roddy, Leah J. Siskind, Paul J. Nietert, Tamara K. Nowling

**Affiliations:** 1 Division of Rheumatology and Immunology, Department of Medicine, Medical University of South Carolina, Charleston, South Carolina, United States of America; 2 Division of Nephrology, Department of Medicine, Medical University of South Carolina, Charleston, South Carolina, United States of America; 3 Department of Public Health Sciences, Medical University of South Carolina, Charleston, South Carolina, United States of America; 4 Department of Drug Discovery and Biomedical Sciences, Medical University of South Carolina, Charleston, South Carolina, United States of America; 5 Research Service, Ralph H. Johnson Veterans Affairs Medical Center, Charleston, South Carolina, United States of America; Beth Israel Deaconess Medical Center, United States of America

## Abstract

Systemic Lupus erythematosus (SLE) is an autoimmune disease caused, in part, by abnormalities in cells of the immune system including B and T cells. Genetically reducing globally the expression of the ETS transcription factor FLI1 by 50% in two lupus mouse models significantly improves disease measures and survival through an unknown mechanism. In this study we analyze the effects of reducing FLI1 in the MRL/lpr lupus prone model on T cell function. We demonstrate that adoptive transfer of MRL/lpr *Fli1* ^*+/+*^ or *Fli1* ^*+/-*^ T cells and B cells into *Rag1*-deficient mice results in significantly decreased serum immunoglobulin levels in animals receiving *Fli1* ^*+/-*^ lupus T cells compared to animals receiving *Fli1* ^*+/+*^ lupus T cells regardless of the genotype of co-transferred lupus B cells. *Ex vivo* analyses of MRL/lpr T cells demonstrated that *Fli1* ^*+/-*^ T cells produce significantly less IL-4 during early and late disease and exhibited significantly decreased TCR-specific activation during early disease compared to *Fli1* ^*+/+*^ T cells. Moreover, the *Fli1* ^*+/-*^ T cells expressed significantly less neuraminidase 1 (*Neu1*) message and decreased NEU activity during early disease and significantly decreased levels of glycosphingolipids during late disease compared to *Fli1* ^*+/+*^ T cells. FLI1 dose-dependently activated the *Neu1* promoter in mouse and human T cell lines. Together, our results suggest reducing FLI1 in lupus decreases the pathogenicity of T cells by decreasing TCR-specific activation and IL-4 production in part through the modulation of glycosphingolipid metabolism. Reducing the expression of FLI1 or targeting the glycosphingolipid metabolic pathway in lupus may serve as a therapeutic approach to treating lupus.

## Introduction

Systemic lupus erythematosus (SLE) is an autoimmune disease characterized by widespread inflammation, autoantibody production, and immune complex deposition. SLE affects nearly every organ system in the body. The underlying cause of SLE is not known but abnormalities in both B and T cells are thought to contribute to the loss of self-tolerance, production of autoantibodies, and deposition of immune complexes in the kidneys and other target tissues. In SLE, B cells demonstrate deregulated cell signaling leading to increased B cell activation and disturbed B cell homeostasis [1–3]. T cells in SLE show aberrant cell signaling, altered gene expression and cytokine production, and increased infiltration into tissues (Reviewed in [4]). Efforts to improve SLE treatment therapies are ongoing but are limited by the lack of understanding of SLE pathogenesis and the specific alterations that occur in the cell types involved.

Friend leukemia virus integration 1 (FLI1), an ETS family transcription factor, plays a role in SLE disease progression as demonstrated in two different lupus mouse models [5,6]. FLI1 is required for embryogenesis and is expressed in the adult thymus, heart, muscle, spleen, lung, and ovary [7]. In the immune system, FLI1 is expressed in immature and mature B cells and throughout T cell development [8–12]. Global overexpression of FLI1 in otherwise healthy mice resulted in development of a lupus-like kidney disease and expansion of autoreactive T cells [13], suggesting a role for FLI1 in lupus disease development/progression. Genetic reduction of FLI1 expression by 50% (*Fli1* ^*+/-*^) in NZM2410 and MRL/lpr lupus prone mice significantly decreased autoantibody levels, renal pathology, and proteinuria and significantly increased survival in comparison to lupus mice with wild-type FLI1 levels (*Fli1* ^*+/+*^) [5,6,14]. Transplantation of bone marrow from MRL/lpr mice with reduced FLI1 expression into irradiated MRL/lpr mice was sufficient to improve disease measures and prolong survival [14] similar to global FLI1 reduction, indicating that reducing FLI1 expression in hematopoietic cells improves lupus disease.

The hematopoietic cell lineage(s) responsible for the protective effect of reducing FLI1 levels in the bone marrow study and the mechanisms involved are unknown. Reducing FLI1 levels in the MRL/lpr and lupus mouse model resulted in an increase in the percentage of naïve T cells [5]. However, the specific effects of reducing FLI1 on lupus T cell function in disease pathogenesis remain uninvestigated. Using an adoptive transfer approach, we demonstrate that transfer of *Fli1* ^*+/-*^ T cells from MRL/lpr mice decreases immunoglobulin production by co-transferred *Fli1* ^*+/+*^ or *Fli1* ^*+/-*^ MRL/lpr B cells. We present data that these effects may be due in part to decreased TCR-specific activation, decreased IL-4 production and altered glycosphingolipid metabolism in the *Fli1* ^*+/-*^ T cells. These novel observations provide important mechanistic insight into the impact of FLI1 levels on lupus T cell function and progression of disease.

## Materials and Methods

### Ethics statement and mouse strains

All animal experiments and methods of euthanasia were approved by the Ralph H. Johnson VAMC Institutional Animal Care and Use Committee (IACUC). Mice were housed and maintained under pathogen-free conditions at the Ralph H. Johnson VAMC Animal Care Facility (Charleston, SC). B6.129S7-Rag1 (*Rag1* ^*-/-*^) mice were obtained from Jackson Laboratories (Bar Harbor, ME). MRL/lpr *Fli1* ^*+/+*^ and *Fli1* ^*+/-*^ mice [5] were obtained from matings between MRL/lpr *Fli1* ^*+/+*^ and MRL/lpr *Fli1* ^*+/-*^ mice in our colony. Age-matched animals of both genders were used in experiments.

### Isolation of T and B cells and T cell stimulations

T and/or B cells were isolated from mouse spleens by gently homogenizing the organ in phosphate buffered saline (PBS), lysing red blood cells (Lonza, Basel, Switzerland) and purifying untouched lymphocyte populations by negative selection using the Pan T cell and B cell Isolation Kits (Miltenyi, Cologne, Germany). Isolated cell populations were analyzed by flow cytometry and were 90-95% pure. The pan T cell kit uses B220 to remove B cells, which also removes the CD3^+^CD4^-^CD8^-^B220^+^ (double negative) T cell population that accumulates in the MRL/lpr model as disease progresses. Flow cytometry analysis of our isolated T cell populations demonstrate that, on average, less than 6% of the T cells that were analyzed in our studies were double negative T cells.

For stimulations, T cells were plated at 1x10^6^ cells per well on a 24-well plate in 1 ml RPMI1640 (Corning Cellgro, Corning, NY) supplemented with 10% fetal bovine serum (FBS) and 1% penicillin/streptomycin solution (Sigma, St. Louis, MO). TCR-specific T cell stimulations were performed using anti-CD3/CD28 conjugated beads from the mouse T cell Activation/Expansion kit (Miltenyi, Cologne, Germany) at a 1:1 bead:cell ratio following the manufacturer’s instructions. T cell stimulation by Phorbol 12-Myristate 13-Acetate (PMA) and ionomycin (ion) (Sigma, St. Louis, MO) were performed using a final concentration of 10 ng/ml PMA and 100 ng/ml ion.

### Adoptive transfer of MRL/lpr T cells and B cells to RAG-1^-/-^

T and B cells were isolated as described above from spleens of two mid-disease stage (14-15 week-old) MRL/lpr *Fli1* ^*+/+*^ and *Fli1* ^*+/-*^ mice, combined, resuspended in 1X PBS and transferred into *Rag-1* ^*-/-*^ mice. 3x10^6^ T and 3x10^6^ B cells were combined for a total of 6x10^6^ cells per recipient mouse and injected into the tail vein of 10 week-old *Rag1* ^*-/-*^ female mice as follows: group 1, *Fli1* ^*+/+*^ T and *Fli1* ^*+/+*^ B cells; group 2, *Fli1* ^*+/-*^ T cells and *Fli1* ^*+/+*^ B cells; group 3, *Fli1* ^*+/-*^ T and *Fli1* ^*+/-*^ B cells; and group 4, *Fli1* ^*+/+*^ T cells and *Fli1* ^*+/-*^ B cells. Two and four weeks after transfer, blood was collected from each *Rag1* ^*-/-*^ recipient, serum prepared and stored at -80°C. Three *Rag1* ^*-/-*^ recipient mice were used for each treatment group per experiment and the experiment was performed twice. Results from both experiments were combined and analyzed as described below.

### Serum anti-dsDNA, anti-GBM, and immunoglobulins

Levels of anti-dsDNA, anti-glomerular basement membrane (GBM) antibodies, and total IgG, IgM, and IgA were measured in mouse serum by ELISA. Measurements were performed as described previously [15–17]. Briefly, 96-well plates were coated overnight with calf thymus DNA (Sigma, St. Louis, MO) in sodium salt citrate buffer for anti-dsDNA, rabbit GBM in PBS for anti-GBM, or goat anti-mouse IgG, IgM, or IgA (Southern Biotechnology Associates, Birmingham, AL). To block, 1% BSA was added to each well and washed with PBS-tween. Sera were added to wells in serial dilutions and incubated for 1 hour at room temperature. After washing, HRP-conjugated secondary antibodies (Sigma, St. Louis, MO) were added and incubated at room temperature. Substrate containing 3,3’,5,5’ – tetramethylbenzidene (TMB, Sigma, St. Louis, MO) in citrate buffer (pH 4.0) and H_2_O_2_ was added for color development. Optical density was measured at A_380_ using a microtitre plate reader (Dynatech, McLean, VA). Serum from individual animals was analyzed and relative levels from both experiments were averaged. The average is presented and “n” = total number of animals analyzed.

### Cytokine level measurements

Cytokine ELISAs for IL-4 and IL-13 (eBioscience, San Diego, CA) were performed following manufacturer’s instructions on media from isolated T cells cultured in the absence or presence of anti-CD3/CD28 conjugated beads for 24 hours at 37°C, 5% CO_2_. Optical density was measured at A_450_ using a BioTek ELx800 microtitre plate reader (BioTek, Winooski, VT). Levels of each cytokine were calculated from standard curves generated from the standards supplied with the kits. T cells from individual animals were analyzed and averaged for each genotype. Relative average levels are presented. “n” = the number of animals analyzed.

### Cell death analyses

Isolated T cells were cultured at 1x10^6^ cells per well in 1 ml media on a 24-well plate in the absence or presence of anti-CD3/CD28 conjugated beads for 3 or 24 hours at 37°C, 5% CO_2_. Cells were collected and labeled with AnnexinV-FITC and propidium iodide (PI) following kit instructions (BD Biosciences, San Jose, CA). Live (Annexin V, PI negative), dead (all PI positive) and apoptosing (Annexin V positive, PI negative) cells were measured on a FACSCalibur cytometer by the Flow Cytometry and Cell Sorting Facility (MUSC). T cells from individual animals were analyzed and averaged for each genotype. Average percentages are presented. “n” = the number of animals analyzed.

### Ca^2+^ influx experiments

T cells were isolated as described above and calcium (Ca^2+^) influx measured essentially as described previously [18] using a Fluorescence Imaging Plate Reader (FLIPR^tetra^) (Molecular Devices, Sunnyvale, CA) at the FLIPR^tetra^ Resource Facility (MUSC). Briefly, isolated T cells were suspended in Hanks Balanced Salt Solution (HBSS) supplemented with 20mM HEPES and 0.2% BSA. Cells were labeled with Calcium5 dye according to manufacturer’s instructions (Molecular Devices, Sunnyvale, CA); after addition of dye, cells were plated at 0.5x10^6^ cells in 100 µl per well on a 96-well plate and incubated at 37°C for 30 minutes. A signal test of the labeled cells was performed to adjust the basal fluorescence of the unstimulated cells between 8,000 and 10,000 units. Ca^2+^ influx following TCR-specific stimulation was measured as follows. Anti-CD3/CD28 conjugated beads (or unloaded beads as control) in 50 µl of supplemented HBSS media were added per well and immediately placed on the FLIPR^tetra^. Fluorescent readings as a measure of Ca^2+^ levels were recorded every second for the first minute and every 5 seconds thereafter for a total of 45 minutes. Ca^2+^ influx following calcium mobilization by the Ca^2+^ ionophore ionomycin was measured as follows. The 96-well plate containing the dye-loaded cells was placed on the FLIPR^tetra^ and fluorescent readings taken every second for five seconds at which time a 3X solution of PMA and ionomycin (PMA/ion) in 50 µl supplemented HBSS was added per well (or buffer only as control) by the FLIPR. Fluorescent readings continued to be recorded every second after addition of PMA/ion for a total of 1 minute. T cells isolated from individual animals were analyzed in triplicate wells and averaged for both experimental and control wells. Average readings from cells in the presence of unloaded beads and buffer only were subtracted from the anti-CD3/CD28-conjugated beads and PMA/ion readings, respectively. The presented data is the average for each genotype. “n” = total number of animals analyzed.

### Cell lines, plasmids and transfections

The Jurkat human T cell line and S1A. TB.4.8.2 (S1A) mouse T cell line were obtained form American Type Culture Collection (Manassas, VA) and maintained as described previously [19,20]. The FLI1 expression plasmid (pcDNA FLI1) was described previously [20]. The human *NEU1* promoter/reporter construct (pSG h*NEU1*) and promoterless reporter construct (pSG Empty) were obtained from Switchgear Genomics (Menlo Park, CA). The pSG h*NEU1* construct contains 968 bp of the proximal human *NEU1* promoter driving Renilla luciferase expression. The mouse *Neu1* P/R construct was generated by amplifying the -3535 to -11 bp region (relative to the translation start site) of the *Neu*1 gene from a mouse BAC clone (Riken DNA Bank, Tokyo, Japan) and ligating into the promoterless pGL3 Basic vector (Promega, Madison, WI). This pGL3 m*Neu1* -3535/-11 construct was sequenced to confirm the presence and correct sequence of *Neu1*. This construct was then digested with KpnI and religated to generate a pGL3 m*Neu1* -435/-11 construct containing the proximal mouse *Neu1* promoter driving Firefly luciferase expression that was used in the transfections. Two individually derived pGL3 m*Neu1* -435/-11 clones were tested and gave similar results.

The pSG h*NEU1* or pSG Empty constructs (0.5 µg) were transfected with the amount of pcDNA FLI1 expression vector indicated in the figure into the Jurkat cell line using Fugene 6 (Promega, Madison, WI) according to manufacturer’s instructions. Jurkat cells were seeded at a density of 1x10^6^ cells per well on a 6-well plate in 2 ml culture media the day of transfection. The pGL3 m*Neu1* or pGL3 Basic constructs (0.5 µg) were transfected with the amount of pcDNA FLI1 expression vector indicated in the figure into the mouse S1A T cell line by nucleofection (Lonza, Basel, Switzerland) as described previously [19,20]. Total molar amount of DNA was kept constant from well to well by addition of empty pcDNA3. Transfection efficiency was normalized by addition of 0.1 µg pGL3 Control vector (Promega, Madison, WI) containing the SV40 promoter driving Firefly luciferase expression for the *hNEU1* transfections and 0.1 µg pRL-TK vector (Promega, Madison, WI) containing the thymidine kinase promoter driving Renilla luciferase expression for the m*Neu1* transfections. Cells were cultured overnight at 37°C, 5% CO_2_ and then in the absence or presence of PMA/ion as described above for an additional 24 hours. Cells were harvested and Firefly luciferase and Renilla luciferase expression measured using the Dual Luciferase Reporter Assay system (Promega, Madison, WI). Each transfection was performed in duplicate and transfections were performed four times with similar results. Results from representative transfections are presented.

### Glycosphingolipid analyses

Isolated T cells were cultured in the absence or presence of anti-CD3/CD28 conjugated beads for 24 hours at 37°C, 5% CO_2_. Cells were pelleted and flash frozen. Lactosylceramide (LacCer) and Glucosylceramide (GluCer) levels were measured by Supercritical Fluid Chromatography (SFC) coupled with tandem mass spectrometry (SFC/MS/MS) by the Lipidomics Core Facility (MUSC) and normalized to cell number. T cells from individual animals were analyzed and then averaged for each genotype. Average levels are presented. “n” = the number of animals analyzed.

### Real-time RT-PCR assays

RNA was prepared from isolated T cells using the RNeasy kit (Qiagen, Hilden, Germany) following manufacturer’s directions and cDNA generated using 0.5-1 µg RNA using the iScript cDNA Synthesis kit (BioRad, Hercules, CA). Real-time PCR was performed with the cDNA using the Lightcycler 480 SYBR Green I Master kit and Lightcycler 480 II (Roche, Indianapolis, IN). Primers used for real-time PCR include: *Neu1*, 5’ ATGTGCCTGTGCTACTCCTG3’ and 5’ GGTCTTGATGATGTCCTTGATGG3’; *Neu3*, 5’ TTCCACCTTCCCTTCCTCATCC3’ and 5’ GCAATAAGCACCGTTATCAACCATG3’; *βactin*, 5’ AGATTACTGCTCTGGCTCCTAG3’ and 5’ CCTGCTTGCTGATCCACATC3’. Relative message levels of *Neu1* and *Neu3* were calculated using the ΔΔCT method, normalizing to βactin levels. The ΔΔCT from one MRL/lpr *Fli1* ^*+/+*^ mouse was set to one and all other mice compared to that mouse. “n” = the number of animals analyzed.

### Neuraminidase activity

T cells were isolated from spleen as indicated above and remained unstimulated or were stimulated in the presence of anti-CD3/CD28 conjugated beads as indicated above for 24 hours. Cells were collected and protein extracts prepared by disruption of cells in protein assay buffer (20 mM Tris-HCl, pH 7.4, 12 mM CaCl_2_, protease inhibitor mix). Protein concentration was measured using the micro-BCA Protein Assay kit (Pierce/Thermo Scientific, Rockford, IL). Total protein was assayed for NEU activity using the Amplex Red Neuraminidase Assay kit (Life Technologies, Grand Island, NY) following manufacturer’s instructions. Absorbance readings as a measure of activity were read at A_540_ using a BioTek ELx800 microtitre plate reader (BioTek, Winooski, VT). T cells from individual animals were analyzed and averaged for each genotype. Relative average levels are presented. “n” = the number of animals analyzed.

### Statistical Analyses

For all assays, each experiment included T cells from 3–5 *Fli1* ^*+/+*^ and 3-5 *Fli1* ^*+/-*^ animals, which were analyzed independently and each experiment was performed 3-4 times. Data from each experiment were averaged for a total of 7-18 animals for each genotype (“n”, as indicated in the figures). In some cases, relative levels were calculated within each experiment due to variability from assay to assay (cytokine, IgG/IgM and NEU activity assays) and the average relative values are presented. For those assays, relative values were used to determine statistically significant differences.

All statistical analyses were conducted using SAS v 9.3 (Cary, NC). For the adoptive transfer data, relative total immunoglobulin levels were compared among the different mouse genotype groups using analysis of variance (ANOVA) models. In the *Rag1* ^*-/-*^ transfer experiment, levels of IgG and IgM were in the range of 0-2 µg/ml for IgM and 10-220 µg/ml for IgG for one experiment and 2-15 µg/ml for IgM and 5-104 µg/ml for IgG for the second experiment. These differences were likely due to disease variability of the MRL/lpr donor mice for each experiment. In order to combine data from both transfers for statistical analyses, relative values were calculated. The observed value for each mouse within an experiment was divided by the average value of group 1. Relative values from both experiments were used in pairwise comparisons between the individual groups using Tukey’s honestly significant difference tests [21].

For the Ca^2+^ influx experiments, general linear mixed models (GLMMs) were constructed to compare the relative light units (RLU) associated with Ca^2+^ influx in MRL/lpr *Fli1* ^*+/+*^ and *Fli1* ^*+/-*^ T cells following stimulation over time. In each of these longitudinal models, fixed effects for time and group (*Fli1* ^*+/+*^ vs. *Fli1* ^*+/-*^) were included, along with quadratic effects for time, and an interaction between group and time. For each model, the interaction between the quadratic time effect and group was not significant and was thus not included in the final model. In addition, the GLMMs included random intercepts and slopes for each mouse, and autoregressive error covariance structures were used to account for the correlation among data points observed within the same mice over time. The analyses for anti-CD3/CD28 stimulation were restricted to data collected after the 60-second time point, since the curves for each of the samples were relatively flat until that time point. Similarly, analyses for ionomycin stimulation were restricted to data collected after the time point at which ionomycin was added to the wells.

Statistically significant differences for the real-time PCR, cytokine, NEU activity and glycosphingolipid data were determined by the Student’s t-test with significant p values provided in the figures and/or text. Error bars in figures represent standard error unless otherwise noted in figure legends.

## Results

### Effects of MRL/lpr Fli1^+/+^ and Fli1^+/-^ T cells after adoptive transfer into Rag1^-/-^ mice

FLI1 is expressed in B cells and in both CD4^+^ and CD8^+^ T cells [9]. Therefore, pan T and B cells were used in all of our studies. It is unknown whether FLI1 is expressed in the double negative (CD3^+^CD4^-^CD8^-^) T cells that accumulate during disease in the MRL/lpr model and the percentage of this double negative cell population was unchanged in the MRL/lpr *Fli1* ^*+/-*^ compared to *Fli1* ^*+/+*^ mice. Therefore, as described in the methods section, the double negative T cells were excluded from our T cell isolations and are not included in our analyses. To examine specific functional effects of Fli1^+/-^ compared to *Fli1* ^*+/+*^ lupus T cells, we performed an adoptive transfer experiment using *Rag1* ^*-/-*^ mice as recipients. *Rag1* ^*-/-*^ mice lack mature T and B cells [22] allowing us to examine isolated effects of the adoptively transferred MRL/lpr lymphocytes. T and B cells were isolated from *Fli1* ^*+/+*^ and *Fli1* ^*+/-*^ spleens of 14-15 week-old (mid-disease) MRL/lpr mice and transferred into *Rag1* ^*-/-*^ recipient mice. Cells were mixed in all possible combinations prior to transfer: group 1, *Fli1* ^*+/+*^ T and *Fli1* ^*+/+*^ B cells; group 2, *Fli1* ^*+/-*^ T and *Fli1* ^*+/+*^ B cells; group 3, *Fli1* ^*+/-*^ T and *Fli1* ^*+/-*^ B cells; and group 4, received *Fli1* ^*+/+*^ T and *Fli1* ^*+/-*^ B cells. The presence of T and B cells in the blood of recipient mice was assessed prior to transfer and at 1 and 4 weeks post transfer by flow cytometry by staining for CD3 and CD19, respectively. CD3^+^ T and CD19^+^ B cells were not present in *Rag1* ^*-/-*^ recipients prior to the transfer. CD3^+^ and CD19^+^ cell numbers in blood at one week and in the lymph node, spleen, and blood at four weeks post transfer were similar between the four groups (data not shown).

**Figure 1:**
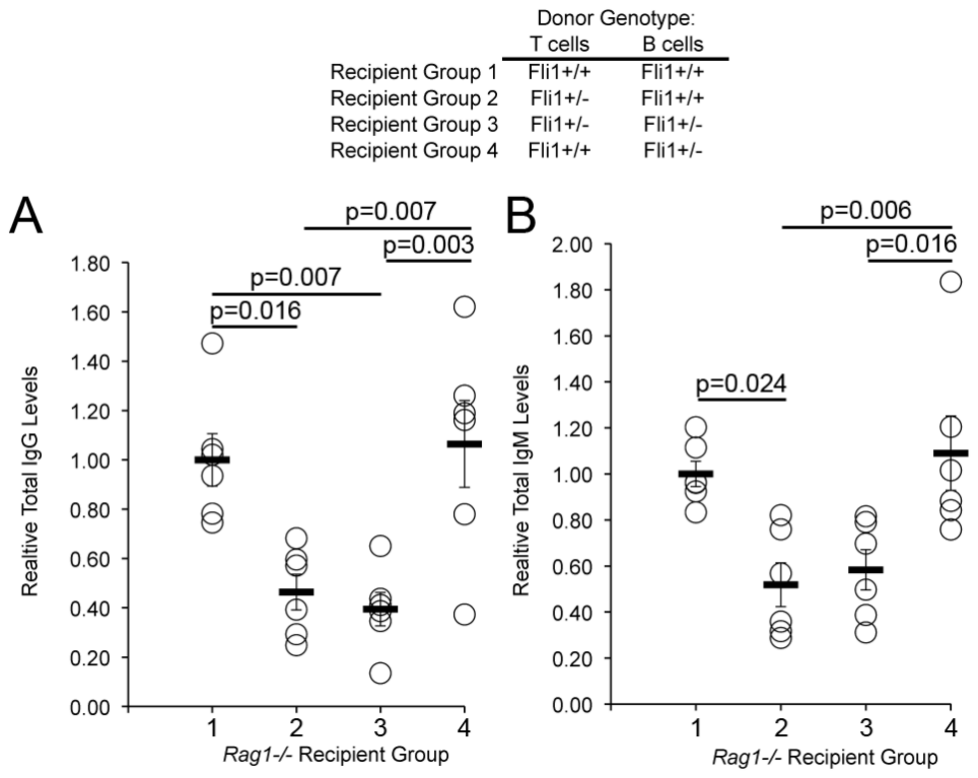
The FLI1 levels in MRL/lpr T cells mediate immunoglobulin production from MRL/lpr B cells when transferred into *Rag-1* ^*-/-*^ mice. MRL/lpr *Fli1* ^*+/+*^ T and B cells and *Fli1* ^*+/-*^ T and B cells were transferred into *Rag1* ^*-/-*^ mice in all possible combinations. Genotypes of MRL/lpr cells transferred into *Rag1* ^*-/-*^ mice are indicated above the graphs: Group 1, *Fli1* ^*+/+*^ T and *Fli1* ^*+/+*^ B cells; Group 2, Fli1^+/-^ T and *Fli1* ^*+/+*^ B cells; Group 3, *Fli1* ^*+/-*^ T and *Fli1* ^*+/-*^ B cells; and Group 4, *Fli1* ^*+/+*^ T and *Fli1* ^*+/-*^ B cells (n=6 for each group). IgG (A) and IgM (B) levels were measured by ELISA in serum collected 2 and 4 weeks after transfer. Relative levels were calculated as indicated in the methods. Data for the 2-week time point are presented and p values are provided within the figure.

Prior to transfer, serum levels of IgG, IgA, IgM, anti-GBM, and anti-dsDNA were measured by ELISA in the *Rag1* ^*-/-*^ recipients and in the MRL/lpr *Fli1* ^*+/+*^ and *Fli1* ^*+/-*^ donors for assessment of disease. Prior to transfer, serum Ig and anti-dsDNA levels were undetectable in *Rag1* ^*-/-*^ mice, in agreement with previously published data [22], and readily detectable in the 14-15 week-old MRL/lpr donor mice with levels being similar in the *Fli1* ^*+/+*^ and *Fli1* ^*+/-*^ mice at this disease stage (data not shown) as reported previously [5]. Anti-GBM, and anti-dsDNA remained undetectable in *Rag1* ^*-/-*^ sera at 2 and 4 weeks after adoptive transfer (data not shown). Results of ANOVA models indicated significant differences between groups with respect to relative IgG levels at week 2 (p=0.001, Figure 1A). Similarly, there were significant differences between groups with respect to IgM at week 2 (p=0.002, Figure 1B). At week 2, pairwise analyses between groups indicated that there were significant differences in relative IgG levels between group 1 (*Fli1* ^*+/+*^ T, *Fli1* ^*+/+*^ B) and group 2 (*Fli1* ^*+/-*^ T, *Fli1* ^*+/+*^ B) (p=0.016), between group 1 and group 3 (*Fli1* ^*+/-*^ T, *Fli1* ^*+/-*^ B) (p=0.007), between group 2 and group 4 (Fli1^+/+^ T, *Fli1* ^*+/-*^ B) (p=0.007), and between group 3 and group 4 (p=0.003) (Figure 1A). Similarly, there were significant differences in relative IgM levels at week 2 between group 1 and group 2 (p=0.024), between group 2 and group 4 (p=0.006), and between group 3 and group 4 (p=0.016) (Figure 1B). The overall levels of IgG and IgM were decreased 4 weeks after transfer and was likely due to a decrease in the surviving numbers of transferred cells. The differences observed 2 weeks after transfer remained at 4 weeks after transfer, but the differences were no longer statistically significant (data now shown). These data demonstrate that the FLI1 levels in MRL/lpr T cells significantly influence IgG and IgM production by co-transferred MRL/lpr B cells regardless of FLI1 levels in the B cells when transferred into *Rag1*-deficient mice.

### Reduction of Fli1 levels in MRL/lpr mice decreases IL-4 production and reduces TCR-specific activation of T cells

**Figure 2:**
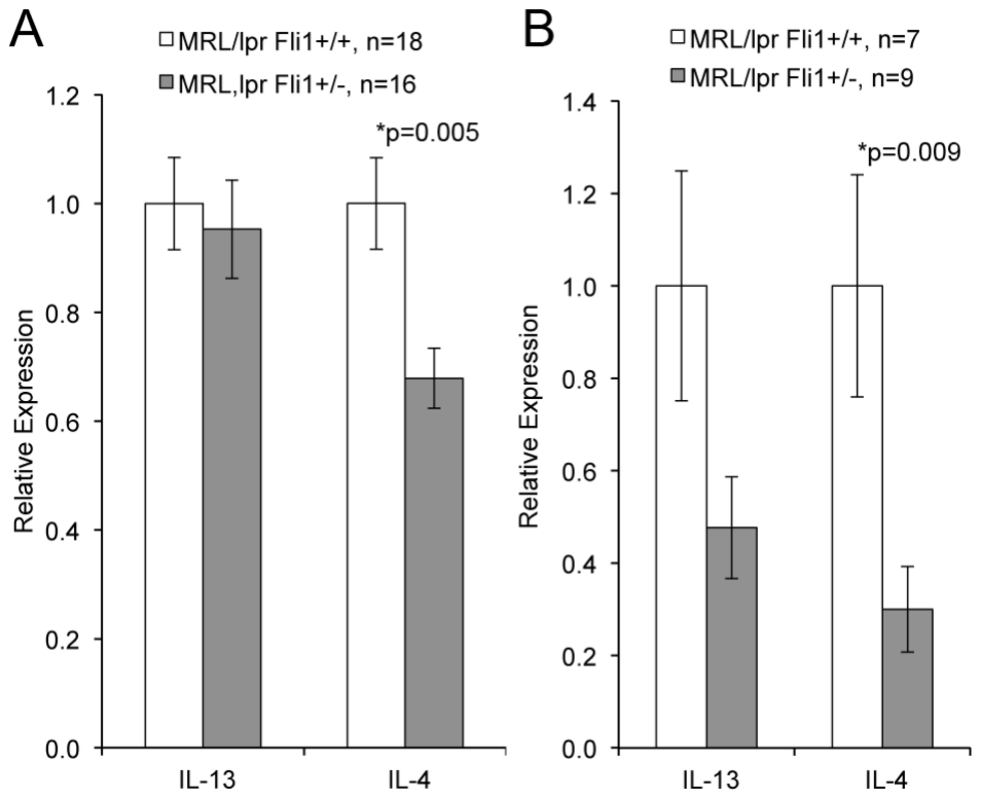
MRL/lpr *Fli1* ^*+/-*^ T cells produce significantly less IL-4 compared to MRL/lpr *Fli1* ^*+/+*^ T cells. T cells were isolated from the spleens of MRL/lpr *Fli1* ^*+/+*^ and *Fli1* ^*+/-*^ 10-12 week-old (A) 17-18 week-old (B) mice and left unstimulated or stimulated with anti-CD3/CD28 conjugated beads for 24 hours. Media was analyzed for IL-4 and IL-13 by ELISA. IL-13 and IL-4 ranged from 19–173 pg/ml and 23-222 pg/ml, respectively for the T cells from 10–12 week-old mice and from 27–175 pg/ml and 140-830 pg/ml, respectively for the T cells from 17–18 week-old mice. Measurements from unstimulated T cells were less than 20 pg/ml for both IL-13 and IL-4 and are not presented. Relative levels were calculated to combine all animals across experiments as described in the methods. The ‘n’ represents data from individual animals and p values are provided within the figure.

Based on the reduced IgG production in the *Rag1* ^*-/-*^ recipients that received *Fli1* ^*+/-*^ T cells compared to those that received *Fli1* ^*+/+*^ T cells, we analyzed cytokine production by MRL/lpr *Fli1* ^*+/+*^ and *Fli1* ^*+/-*^ T cells. An initial cytokine bead array showed a decrease in IL-4 production from *Fli1* ^*+/-*^ compared to *Fli1* ^*+/+*^ T cells in 17 week-old mice (data not shown). Th2 cytokines such as IL-4 are important for providing help to autoantibody producing B cells. To confirm the results from the bead array and analyze whether reducing FLI1 levels has specific effects on Th2 cytokine expression, IL-4 and IL-13 cytokine production was measured by ELISA. T cells from early disease stage (10-12 weeks), prior to measurable hallmarks of disease, and late disease stage (17-18 weeks), when disease expression is readily evident, were used in the cytokine analyses. Significantly less IL-4 was produced by early (Figure 2A, p=0.005) and late (Figure 2B, p=0.009) disease MRL/lpr *Fli1* ^*+/-*^ compared to *Fli1* ^*+/+*^ T cells from age-matched mice. Less IL-13 production also was observed from late disease *Fli1* ^*+/-*^ compared to *Fli1* ^*+/+*^ T cells, but the differences did not reach statistical significance.

**Figure 3:**
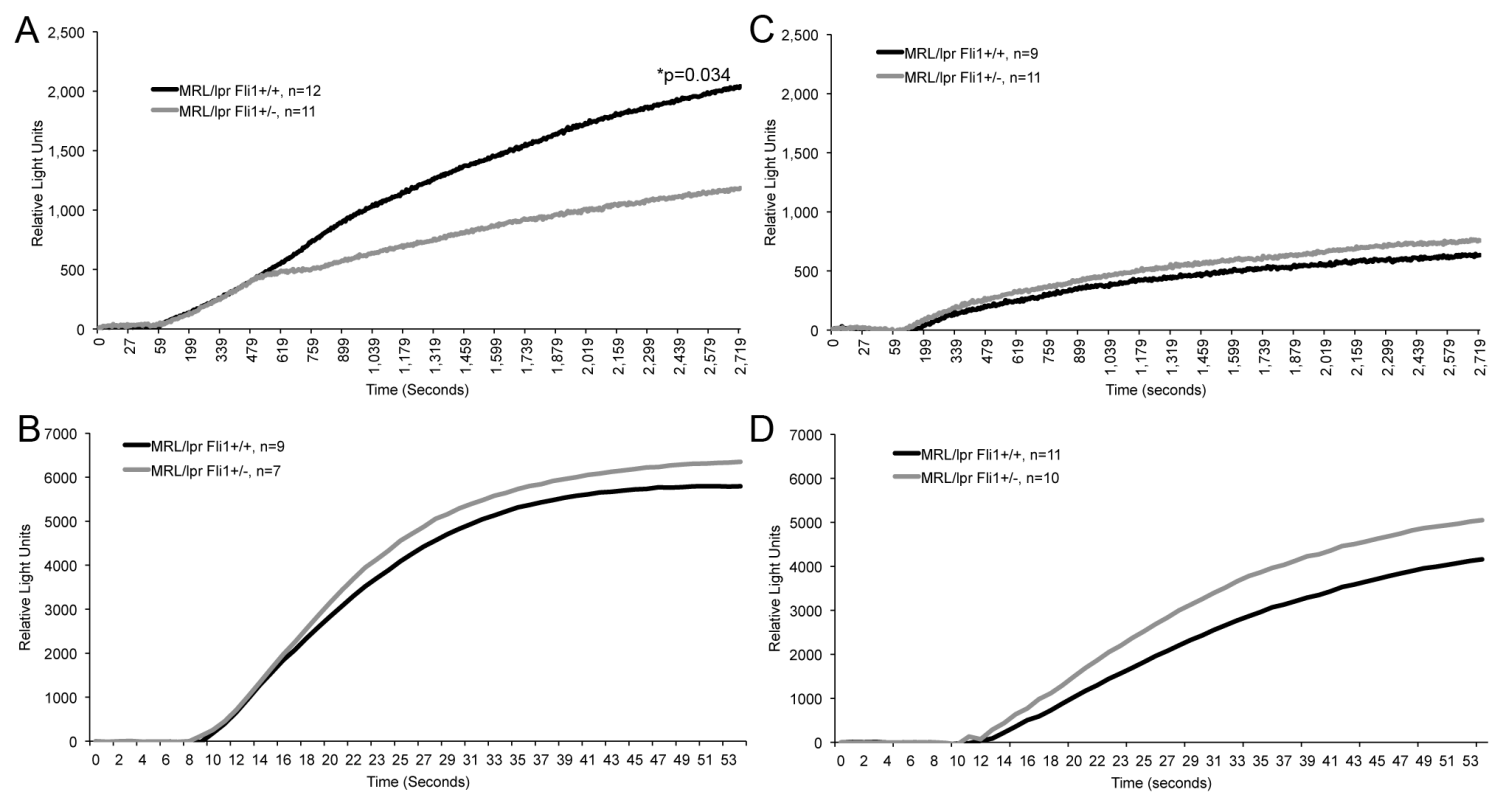
Intracellular Ca^2+^ levels are significantly reduced in *Fli1* ^*+/-*^ T cells compared to *Fli1* ^*+/+*^ T cells from early disease stage mice following TCR-specific activation. T cells from the spleens of MRL/lpr *Fli1* ^*+/+*^ and *Fli1* ^*+/-*^ 10-12 week-old (A and B) and 17-18 week-old (C and D) mice were stimulated with anti-CD3/CD28 (A and C) or PMA/ionomycin (B and D) and Ca^2+^ influx measured over time. PMA/ion was added 5 seconds after beginning data collection and data was collected for 1 min. Anti-CD3/CD28 antibodies were added just before beginning data collection and data was collected for 45 minutes. All data collection points are presented from 0–60 sec for PMA/ion and 0-45 minutes for anti-CD3/CD28. The ‘n’ represents data from individual animals and p values are provided within the figure.

Lupus T cells have altered T cell receptor (TCR)-signaling [23–26]. To examine whether FLI1 levels have an effect on T cell activation, intracellular calcium (Ca^2+^) levels were analyzed in T cells from early and late disease stage mice in real-time following *ex vivo* stimulation. The unadjusted average RLUs over time as a measure of intracellular Ca^2+^ in response to stimulation by anti-CD3/CD28 and the calcium ionophore ionomycin are illustrated in Figure 3A and 3B, respectively for the T cells from early disease stage mice. In the longitudinal analyses for Ca^2+^ levels in response to anti-CD3/CD28, there was a significant (p=0.034) interaction between group and time, indicating that the average Ca^2+^ influx over time among the *Fli1* ^*+/+*^ group was significantly higher than that among the *Fli1* ^*+/-*^ group. By the end of the experiment the levels in the *Fli1* ^*+/+*^ group were 902 units higher, on average, (95% confidence interval: 64 to 1741) than in the *Fli1* ^*+/-*^ group. Data from one mouse were not included in the anti-CD3/CD28 analyses since it exhibited an extreme degree of experimental variability. As a sensitivity analyses, a similar model was constructed that included data from the originally excluded mouse, and although the statistical significance was slightly attenuated (p=0.11), the magnitude and direction of the longitudinal group differences remained largely unchanged. The significant difference in Ca^2+^ flux following anti-CD3/CD28 stimulation was not observed in T cells from the late disease stage mice (Figure 3C). Interestingly, the Ca^2+^ flux in both the *Fli1* ^*+/+*^ and *Fli1* ^*+/-*^ T cells from the late disease stage mice was 1.5-fold to 2.5-fold lower (Figure 3A vs 3C). In the longitudinal GLMM for Ca^2+^ levels in response to ionomycin, there was no significant interaction between group and time and no significant effect for group, indicating that the average trajectories over time in the *Fli1* ^*+/+*^ and *Fli1* ^*+/-*^ groups were similar (Figure 3B and 3D). These results suggest that FLI1 levels have an effect on TCR-specific activation.

**Figure 4:**
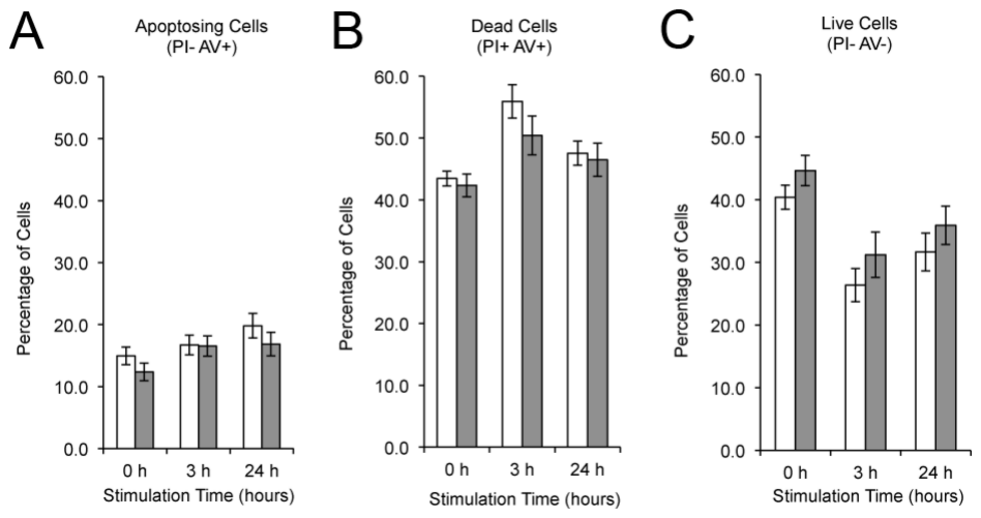
FLI1 levels have no effect on cell death of MRL/lpr T cells following TCR-specific activation. T cells from the spleens of MRL/lpr *Fli1* ^*+/+*^ and *Fli1* ^*+/-*^ 10-12 week-old mice were stimulated with anti-CD3/CD28 for 0, 3 and 24 hours and cell death measured by flow cytometry following Annexin V and PI staining. A) Annexin V+/PI- (apoptosing cells), B) Annexin V+/PI+ (dead cells), and C) Annexin V-/PI- (live cells). The ‘n’ represents data from individual animals.

To ensure the observed differences in cytokine production and Ca^2+^ influx in the T cells from early disease stage mice were not a result of increased cell death, we measured the number of dead and apoptotic cells by flow cytometry following PI and Annexin V staining. We chose to measure cell death 0h, 3h and 24h after stimulation based on the time points at which FLI1 protein and *Fli1* message is down-regulated (3h) and then re-expressed (24h) following stimulation and within the time frame our functional assays were performed (0-24h). T cells were isolated from early disease stage MRL/lpr *Fli1* ^*+/+*^ and *Fli1* ^*+/-*^ mice and stimulated *ex vivo* with anti-CD3/CD28. No significant differences in cell death were detected in MRL/lpr *Fli1* ^*+/-*^ compared to *Fli1* ^*+/+*^ T cells (Figure 4). Together, these results demonstrate that reducing the levels of FLI1 in MRL/lpr mice significantly decreases the response of T cells to TCR-specific activation and production of IL-4 during early disease with the decrease in IL-4 being maintained during late disease.

### Reduction of FLI1 levels in MRL/lpr mice alters glycosphingolipid metabolism in T cells

**Figure 5:**
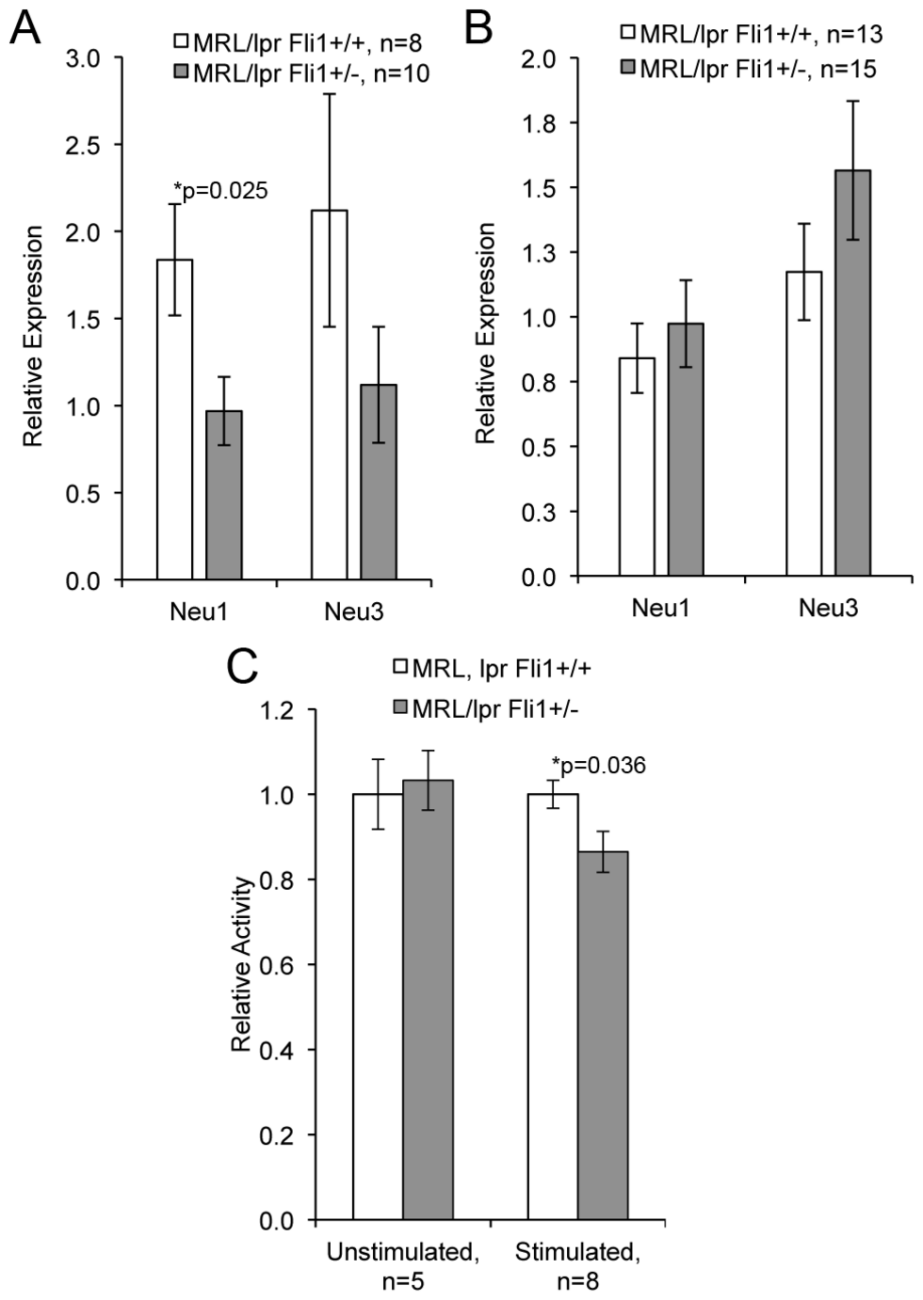
*Fli1* ^*+/-*^ T cells have significantly lower levels of Neuraminidase 1 (*Neu1*) message and NEU activity compared to *Fli1* ^*+/+*^ T cells during early disease. cDNA was amplified from RNA isolated from T cells of MRL/lpr *Fli1* ^*+/+*^ and *Fli1* ^*+/-*^ 10-12 week-old mice (A) and 17-18 week-old mice (C). *Neu1* and *Neu3* message levels were measured by real-time PCR and normalized to *β-actin* levels. B) NEU activity was measured as described in the methods. Relative levels in the NEU activity assay were calculated to combine all animals across experiments as described in the methods. The ‘n’ represents data from individual animals and p values are provided within the figure.

Neuraminidases (NEU) catalyze the breakdown of gangliosides to glycosphingolipids lactosylceramide (LacCer) and glucosylceramide (GluCer), which play a role in the activation of T cells [27,28]. NEU1 is one of four known NEUs, but only NEU1 and NEU3 are expressed in T cells. Specifically, NEU1 is required for TCR-specific activation and IL-4 production of T cells [29,30]. Therefore, we analyzed whether the effects of FLI1 levels on Ca^2+^ influx and IL-4 production may be mediated by a change in NEU1 (or NEU3) expression. Both *Neu1 and Neu3* message levels were decreased in *Fli1* ^*+/-*^ compared to *Fli1* ^*+/+*^ T cells from early disease stage MRL/lpr mice with *Neu1* levels being significantly decreased (p=0.025) (Figure 5A). We also observed a significant decrease in overall NEU enzymatic activity in the *Fli1* ^*+/-*^ compared to *Fli1* ^*+/+*^ stimulated T cells at the early stage disease (Figure 5C; p=0.036). No significant differences in *Neu1* or *Neu3* message levels (Figure 5B) or NEU activity (data not shown) were observed in *Fli1* ^*+/-*^ compared to *Fli1* ^*+/+*^ T cells from late disease stage mice.

**Figure 6:**
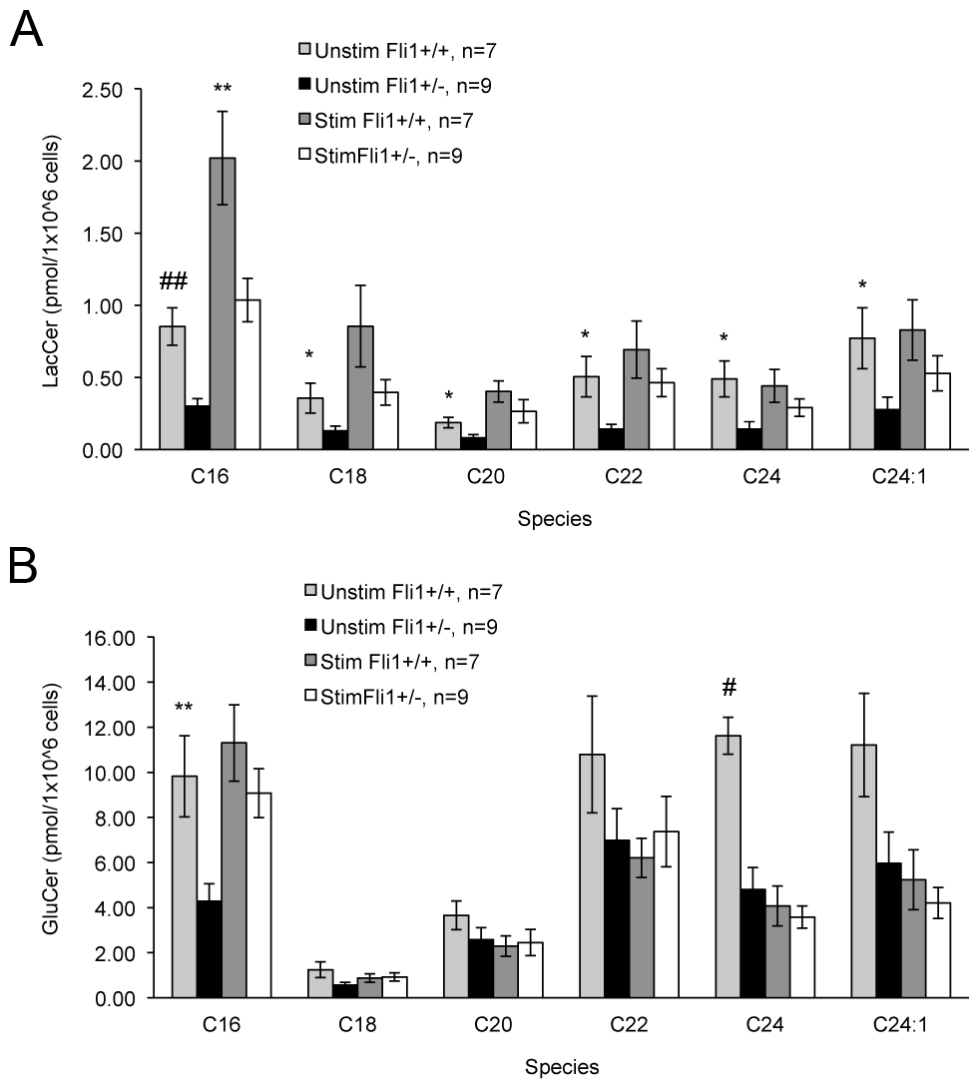
Glycosphingolipid levels are significantly reduced in T cells from late disease stage MRL/lpr *Fli1* ^*+/-*^ compared to MRL/lpr *Fli1* ^*+/+*^ mice. Glycosphingolipids lactosylceramide (LacCer) (A) and glucosylceramide (GluCer) (B) were measured by SFC/MS/MS in unstimulated or anti-CD3/CD28 stimulated T cells isolated from 17–18 week-old MRL/lpr mice. *p<0.05, **p<0.01, #p<0.005, # #p<0.001. The ‘n’ represents data from individual animals.

As another measure of glycosphingolipid metabolism in the late disease stage T cells, we analyzed the levels of LacCer and GluCer. Lipidomic analysis of T cells isolated from late disease stage MRL/lpr *Fli1* ^*+/+*^ and *Fli1* ^*+/-*^ mice revealed a significant reduction in the levels of all the LacCer species (Figure 6A) and major GluCer species C16 and C24 (Figure 6B) in *Fli1* ^*+/-*^ T cells compared to *Fli1* ^*+/+*^ T cells. When these T cells were stimulated *ex vivo* through the TCR, the LacCer species increased, but still tended to remain lower in the *Fli1* ^*+/-*^ T cells compared to the *Fli1* ^*+/+*^ T cells with significant differences being maintained in the major LacCer species C16 (Figure 6A). These results further support the data in the T cells from early disease stage mice that FLI1 levels impact glycosphingolipid metabolism.

**Figure 7:**
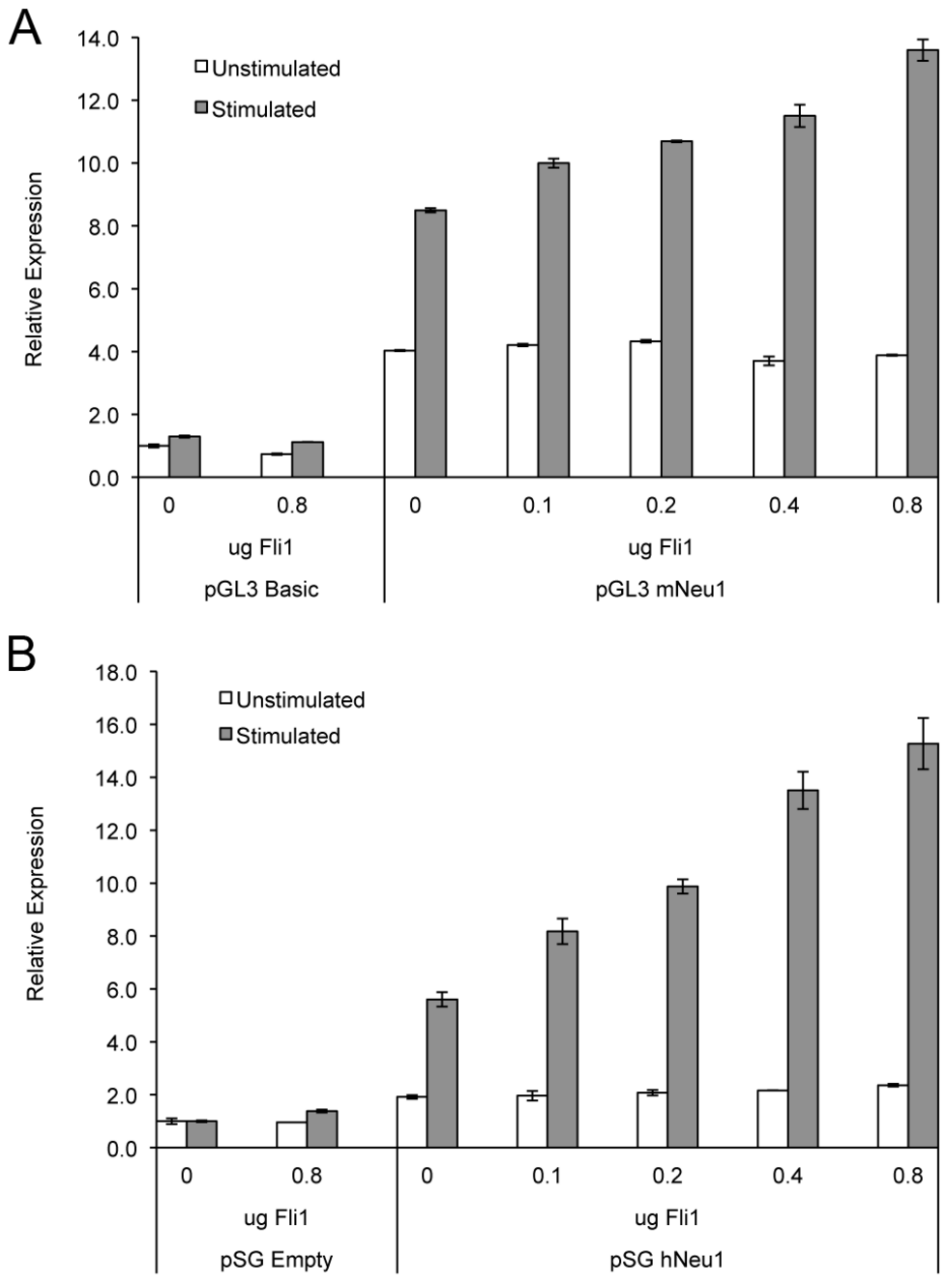
Both the mouse and human *Neu1* promoters respond to FLI1 in a dose-dependent manner. Promoter/reporter constructs pGL3 *mNeu1* containing the mouse promoter (A) or pSG *hNEU1* containing the human promoter (B) were transfected with increasing amounts of a FLI1 expression vector into the mouse S1A (A) or human Jurkat (B) T cell lines. Transfection efficiency was adjusted for by expression from a co-transfected normalization plasmid. Results are representative of four independent transfections with similar results.

To test whether FLI1 can transcriptionally regulate *Neu1* expression in T cells, we co-transfected promoter/reporter constructs containing the mouse *Neu1* or human *NEU1* proximal promoter regions with increasing amounts of a FLI1 expression vector into mouse or human T cell lines, respectively. Both the mouse (Figure 7A) and human (Figure 7B) *Neu1* promoters responded in a dose-dependent manner to FLI1 in stimulated T cells, while the promoterless constructs did not. These results demonstrate that FLI1 can regulate, either directly or indirectly, *Neu1* transcription.

## Discussion

Several studies support a role for T cells as mediators of disease and tissue injury in SLE. Removal of T cells by various methods in different lupus prone mouse models including the MRL/lpr model results in decreased IgG production, decreased nephritis and increased/prolonged survival [31–35]. Lupus patient T cells augment the production of IgG anti-DNA antibodies by autologous B cells *ex vivo* [36]. Clearly, T cells play an important role in autoantibody production by B cells in lupus contributing to progression of disease. Globally reducing the levels of the transcription factor FLI1 in the MRL/lpr and NZM2410 lupus mouse models significantly improves disease by decreasing autoantibody production and increasing survival [5,6,37]. . The mechanism(s) and the cell types involved in the protective effect of reducing FLI1 levels in lupus mice are not completely understood.

Here we show that *Rag1* ^*-/-*^ mice that received *Fli1* ^*+/-*^ (FLI1 levels are 50% of *Fli1* ^*+/+*^ [5]) T cells, regardless of FLI1 levels in co-transferred B cells, had significantly lower IgG and/or IgM levels compared to mice that received *Fli1* ^*+/+*^ T cells. These results suggest that the levels of FLI1 in lupus T cells influence autoantibody production by B cells and that FLI1 plays a role in this T cell function. The presence of FLI1 in both T and B cells argue strongly that the reduced levels in *Fli1* ^*+/-*^ cells influence the function of these cells, especially when examined in the non-autoimmune *Rag1* ^*-/-*^ background. However, the donor cells were isolated from 14–15 week-old MRL/lpr mice, which exhibit disease hallmarks such as measurable proteinuria and autoantibodies. Thus, we cannot rule out the possibility of cell extrinsic effects on the donor cells prior to transfer that may have impacted their function after transfer into the recipients. T cell function of cells isolated from pre/early disease stage is less likely to be influenced extrinsically by disease expression. For this reason, we chose to examine T cells from early-disease (10-12 weeks) and late disease (17-18 weeks) stage MRL/lpr mice for the subsequent analyses.

Based on the effects in our *Rag1* ^*-/-*^ adoptive transfer experiment, we investigated the effects of FLI1 levels on T cell function by measuring cytokine production and TCR-specific activation of MRL/lpr *Fli1* ^*+/-*^ compared to *Fli1* ^*+/+*^ T cells. Our results demonstrated that *Fli1* ^*+/-*^ T cells from early disease stage MRL/lpr mice produced significantly less IL-4 and exhibited reduced TCR-specific activation. Lupus T cells display hyper-responsiveness, a defective proliferative response, defective T cell receptor (TCR)-signaling, increased response to stimulation, and increased resistance to activation-induced apoptosis [23–26]. IL-4 production by T cells is important for the provision of help to autoantibody producing B cells. Global over-expression of IL-4 in the C3H mouse strain resulted in elevated serum IgG levels and autoimmune glomerulonephritis [38] while IL-4 deficient lupus prone mice had improved disease [39]. Our results suggest decreased response to TCR-specific activation and decreased IL-4 production in Fli1^+/-^ T cells may be one mechanism involved in decreasing immunoglobulin production by B cells in the *Rag1* ^*-/-*^ recipient mice and an underlying protective effect of reducing FLI1 levels in lupus prone mice.

We further examined the mechanisms by which FLI1 may be controlling TCR-specific activation and IL-4 production in lupus T cells. In T cells, NEU1 enhances responses to MHCII presentation by B cells [40] and is necessary for TCR-specific calcium influx and IL-4 production [29,30]. The significant reduction in IL-4 and TCR-specific activation in the MRL/lpr *Fli1* ^*+/-*^ T cells during early disease suggested that the neuraminidase NEU1 may mediate these effects. Indeed, we demonstrate that MRL/lpr *Fli1* ^*+/-*^ T cells express significantly less *Neu1* message and have significantly decreased NEU activity at the early disease stage.

In our studies we analyzed pan T cells, but acknowledge that FLI1 may have differential effects in specific T cell subtypes. Future studies are focused on determining whether Fli1 has differential effects in specific subtypes (CD4+ vs CD8+ or Th1 vs Th2 vs Th17 vs Treg). FLI1 is expressed in mature, naïve CD4^+^ and CD8^+^ T cells [9]. FLI1 levels in T cells begin to decrease within 2h of stimulation with near complete turnover occurring by 24h [11]. Fli1 message levels in T cells are down-regulated within 4h of stimulation with levels being restored to pre-stimulation levels by 24 hours after stimulation [8]. Newly synthesized protein levels are undetectable by 1h after stimulation and return to pre-stimulation levels by 48h while turnover of existing protein begins to decline steadily between 2–24h after stimulation [11]. Therefore, FLI1 protein is present in stimulated T cells, but at reduced levels 2-24h after stimulation. NEU1 also is expressed in mature, naïve T cells, but is upregulated upon stimulation at which time it shuttles from the lysosome to the plasma membrane [29,41]. Although a direct comparison between the relative message levels of Neu1 from the early and late disease stage *Fli1* ^*+/+*^ T cells was not shown in Figure 5, we calculated a significant 2.7-fold increase (p=0.013) in *Neu1* message in *Fli1* ^*+/+*^ T cells from late compared to early disease. The increase in *Neu1* message levels at the late disease stage is likely due to the hyperactivated state of the T cells at this stage of disease and agrees with the observation that NEU1 levels are increased when T cells are activated.

Further evidence to support the regulation of *Neu1* expression by FLI1 was demonstrated by co-transfection experiments. The mouse *Neu1* and human *NEU1* promoters responded in a dose-dependent manner to FLI1 in stimulated mouse and human T cells, respectively. We speculate that the levels of FLI1 in the unstimulated (naïve) T cells controls the initial up-regulation of *Neu1* transcription when the cells are stimulated and then may function to maintain expression in the fully activated cells. Together, our results in the T cells from early disease lupus mice suggest FLI1 controls directly or indirectly the transcription of *Neu1* such that reduction of FLI1 leads to decreased Neu1 expression/activity leading to decreased TCR-specific activation and IL-4 production. Of translational interest, the human *NEU1* gene is located on chromosome 6 within the strongest genetic risk factor loci for lupus, the HLA region.

The late disease stage *Fli1* ^*+/-*^ and *Fli1* ^*+/+*^ T cells also showed significantly decreased IL-4 production that may contribute to the observed decrease in autoantibodies in the MRL/lpr model at that stage [5]. However, they had similar calcium responses to TCR stimulation. The 1.5-fold to 2-fold overall lower Ca^2+^ flux in both the *Fli1* ^*+/+*^ and *Fli1* ^*+/-*^ T cells in response to TCR stimulation compared to the early disease stage cells, suggest that extrinsic effects of the disease state (e.g. constant antigenic stimulation) may negate the effects of decreased FLI1 levels on TCR-specific activation observed at the early disease stage. Although the MRL/lpr *Fli1* ^*+/-*^ mice have significantly reduced levels they still have measurable levels of circulating IgG and anti-dsDNA antibodies [5]. The significantly reduced, but still measurable, disease hallmarks of the *Fli1* ^*+/-*^ compared to the *Fli1* ^*+/+*^ MRL/lpr mice at 17-18 weeks of age [5] may impact T cell function extrinsically. Since we did not observe differences in *Neu1* or *Neu3* message levels or NEU activity in the T cells from late disease stage mice we analyzed LacCer and GluCer levels as another measure of change in glycosphingolipid metabolism in these cells. NEU1 functions by removing sialic acids from gangliosides to generate the glycosphingolipid LacCer, which can be further metabolized to GluCer. Glycosphingolipids, along with cholesterol and the TCR complex, compose lipid rafts, which play a central role in TCR signaling and are altered in lupus T cells [42–44] and reducing glycosphingolipid levels in T cells lowers TCR signaling and production of cytokines [28,45]. We observed a significant decrease in LacCer and GluCer species in the late disease stage *Fli1* ^*+/-*^ compared to *Fli1* ^*+/+*^ T cells, suggesting that FLI1 levels do impact glycosphingolipid metabolism in T cells during late disease stage. The mechanisms by which FLI1 controls T cell IL-4 production and mediate glycosphingolipid metabolism at this late disease stage are not clear and will require additional studies to delineate. However, FLI1 does appear to play a role in mediating the glycosphingolipid pathway and warrants further investigation.

In summary, our results demonstrate a role for FLI1 in regulating lupus T cell activation and IL-4 production through modulation of glycosphingolipid metabolism, specifically by mediating the breakdown pathway through the control of *Neu1* expression and/or NEU activity during early disease. Furthermore, our results suggest that the functional effects of reducing FLI1 on lupus T cells may mediate autoantibody production. However, our results in the late disease stage T cells do not rule out the possibility that FLI1 levels also may impact alternate pathways of glycosphingolipid metabolism leading to GluCer and LacCer generation. FLI1 has effects in other cell types, such as B cells [12] and endothelial cells [46], suggesting FLI1 may play an extrinsic role, as well as an intrinsic role, on T cell function. Delineating the extrinsic versus intrinsic effects of altering FLI1 levels in T cells and the contribution of other cell types to the protective effect of reducing FLI1 levels in lupus are ongoing. Importantly, our studies suggest that reducing FLI1 levels and/or inhibiting the glycosphingolipid metabolic pathway, may serve as a therapeutic approach for reducing lupus T cell pathogenesis and treating disease.
